# Correction: Gold–carbonyl group interactions in the electrochemistry of anthraquinone thiols self-assembled on Au(111)-surfaces†Electronic supplementary information (ESI) available: Additional experimental and calculations details. See DOI: 10.1039/c9sc00061e


**DOI:** 10.1039/c9sc90101a

**Published:** 2019-05-09

**Authors:** Michal Wagner, Katrine Qvortrup, Katja E. Grier, Mikkel R. Ottosen, Jonas O. Petersen, David Tanner, Jens Ulstrup, Jingdong Zhang

**Affiliations:** a Department of Chemistry , Technical University of Denmark , Kemitorvet, Building 207 , 2800 Kgs. Lyngby , Denmark . Email: michal.wagner83@gmail.com

## Abstract

Correction for ‘Gold–carbonyl group interactions in the electrochemistry of anthraquinone thiols self-assembled on Au(111)-surfaces’ by Michal Wagner *et al.*, *Chem. Sci.*, 2019, **10**, 3927–3936.



## 

The authors regret that Scheme 1 is incorrect in the original manuscript. The correct scheme is displayed below.

**Scheme 1:**
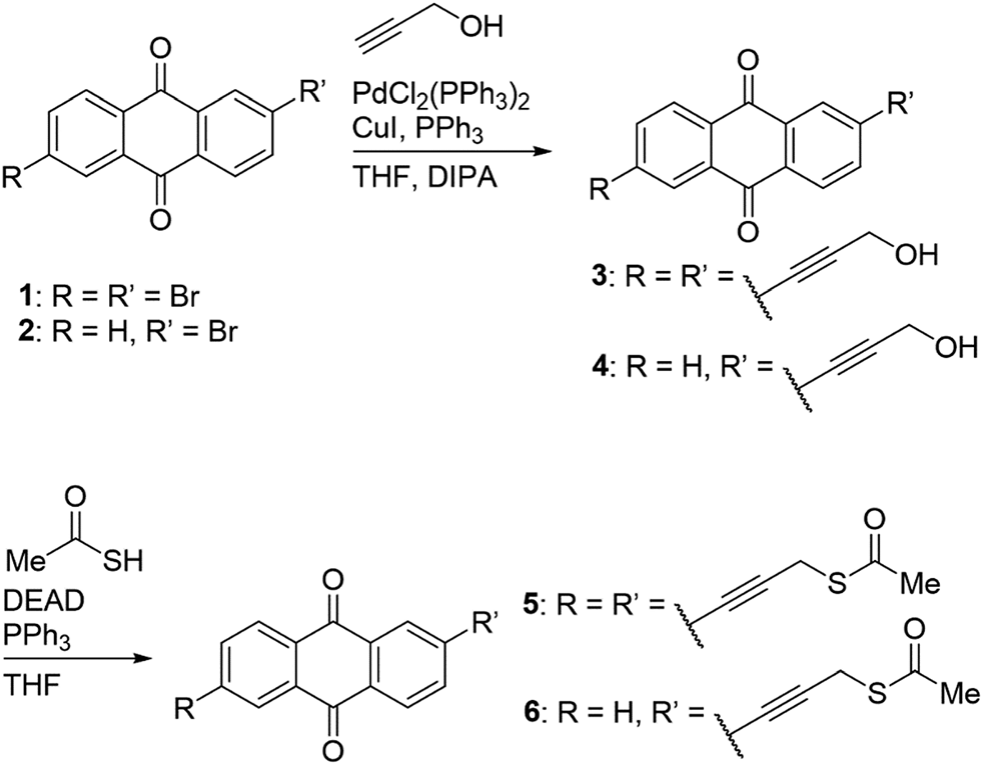
The reactions and resultant chemical structures of AQ2 (**5**) and AQ1 (**6**) compounds.

Additionally, there were errors in the equation notations in the captions of Fig. 7 and 8. The correct figure captions are given below.

Fig. 7 Tafel plots for AQ1- (A) and AQ2-SAMs (B), from CV (up to 20 V s^–1^ scan rate) at pH 4.5. The fitting at low overpotentials is based on eqn (3) and (4) (BV), and the estimation of the reorganization energy is based on fitting of eqn (5) at higher overpotentials (BV*).

Fig. 8 The dependence of normalized transition probability (with respect to *η* = 0 V) and the Fermi function on the electronic energy (A), together with calculated normalized *i*/*η* relationship (B), for selected *λ*-values. The transition probability was calculated using eqn (8), and the *i*/*η* relationships using a reformulation of eqn (6) (eqn (S2)†).

The Royal Society of Chemistry apologises for these errors and any consequent inconvenience to authors and readers.

